# Risk factor management and OCT characteristics of plaque vulnerability: the Holy Grail of plaque and patient vulnerability

**DOI:** 10.1007/s10554-021-02320-1

**Published:** 2021-07-13

**Authors:** J. J. Wykrzykowska, M. P. L. Renkens

**Affiliations:** 1 UMC Groningen, 9713 GZ Groningen, The Netherlands; 2 Amsterdam UMC, 1105 AZ Amsterdam, The Netherlands

The majority of CVD related deaths are caused by coronary artery disease (CAD) and strokes, both phenotypic expressions from significant underlying atherosclerosis [1]. Despite targeting well known risk factors for atherosclerosis such as aging, sex and other risk factors such as smoking, obesity, hypertension, hyperlipidemia, and certainly diabetes still, many of these patient require invasive treatment with percutaneous coronary intervention (PCI), which comes with the price of device thrombosis and restenosis [2].

The latter, remains a major problem with use of modern stents, especially in patients with diabetes mellitus. Patients with diabetes comprise 25–30% of all patients undergoing coronary revascularization and have a two-threefold increased risk for cardiovascular events to occur. Diabetic patients have much higher clinically indicated TLR rates compared to non-diabetic patients even with use of modern stents: 8.6% vs. 5.1% during 2 years of follow-up [3]. If performed in multiple or more complex lesions (SYNTAX score > 11) these rates are even higher: 9.6% vs 6.5% [4–6]. Newly developed devices such as the Cre8 stent and the Abluminus stent promise to overcome this issue but their effectiveness still has to be proven in larger patient populations [7–9]. Therefore, optimal medical therapy is pivotal to synergize the effect of successful PCI on long-term patient outcomes and preventing future events from occurring.

Multiple studies on high intensity statin therapy in patients with established cardiovascular disease have shown to reduce the risk of adverse cardiovascular events by 50–60% [10, 11]. Not only regarding long-term outcomes but also periprocedural [12]. Moreover, statin therapy has also shown a reduction of plaque burden and plaque regression analysed by IVUS imaging [13–15]. The absolute risk reduction in cardiovascular events with LDL-C lowering drugs is greater in patients at higher baseline risk [16]. Serum LDL-C/HDL-C ratio > 2.0 and low Apolipoprotein A1 both seem to be associated with characteristics of vulnerable plaques [17]. In the ATHEROREMO-IVUS study, necrotic core fraction, LCBI and plaque burden are associated with certain elevated molecular lipid serum levels. Fibrous cap thickness was not [18]. Furthermore, the IBIS-4 trial demonstrated regression of coronary atherosclerosis in non-infarct-related arteries in STEMI patients without changes in RF-IVUS defined necrotic core or plaque phenotype after treatment with rosuvastatin 13 months after the index event [19].

For glycemic control, data is a bit less voluminous but a meta-analysis including randomized control trials and registry studies demonstrate a beneficial effect on risk reduction of coronary artery disease [RR 0.89 (95% CI 0.81–0.96)] and nonfatal myocardial infarction [RR 0.84 (95% CI 0.75–0.94)] with intensive glucose lowering therapy [20–22]. Imaging data to show plaque regression or changes in plaque morphology in the coronary arteries with aggressive glycemic control is lacking [23]. In this issue, Ueyama et al. report the results of a single-centre retrospective analysis investigating the relationship between serum hemoglobin A1C (HbA1c) and plaque characteristics as assessed by optimal coherence tomography in 261 patients with de novo stable CAD undergoing PCI [24]. The authors predefined three tertiles of serum HbA1c-levels to compare findings between groups of equal size (tertile 1: HbA1c < 6.3%, tertile 2: 6.3 ≤ HbA1c < 7.8%, tertile 3: HbA1c ≥ 7.8%, each group n = 87). Besides the rates of diabetes, previous CABG and HDL/triglycerides-levels baseline characteristics were comparable between all three groups. With increasing HbA1c-level authors found (1) Fibrous cap (FCT) to be thinner (beta coefficient − 4.89, 95% confidence interval − 8.40 to − 1.39), (2) the prevalence of thin cap fibroadenoma (TCFA) to be increased following an exponential curve (see Figure 4 article) and (3) Minimal lumen area (MLA) and reference lumen to be decreased. OCT characteristics of vulnerable plaques are large plaque burden > 70%, a small lumen area < 4 mm^2^ and high lipid content. These characteristics all represent a lesion which has potential low resistance to mechanical stress forces (e.g. non-laminar flow or wall shear stress).

These important findings emphasize the hypothesis that uncontrolled/not well controlled risk factors may contribute significantly to the progression of atherosclerosis with the formation of vulnerable plaques characteristics. Uncontrolled lipid metabolism might contribute to the formation of an unstable lipid core and uncontrolled glycaemic control might further enhance this effect while at the same time also impairing endogenous plaque sealing through decreased FCT with increased prevalence of TCFA induced by pro-inflammatory cascades. Previous studies have already addressed these OCT plaque characteristics (high plaque burden, high amount of lipid core content, thin fibrous cap, and small MLA) to be associated with Major Cardiac Events when left untreated [25–29].

Altogether this report and previous reports emphasize the pivotal importance of optimal medical therapy for secondary prevention after coronary revascularization to prevent future events caused by the formation of vulnerable plaques. We could hypothesize, however, that different risk factors may be more important in different patients. In diabetic patients glycaemic control may outweigh the importance of LDL suppression. For some patients all risk factors may have to be aggressively managed. One could propose here a paradigm shift to individualized secondary prevention after PCI based on risk factors that are present together with plaque characteristics found during intracoronary imaging. For example, in diabetic patients another type of glucose lowering therapy (SGLT-2 in patients with previous CVD and/or heart failure) may be more important in reducing future events than very aggressive of glycaemic control with risk of hypoglycaemia.

Multicentre studies focussing on patient risk assessment, including the presence of atherosclerotic risk factors and plaque characteristics found in intracoronary imaging, accompanied with adequate long-term follow-up in these patients might be the next step to provide a truthful real world risk assessment of plaque vulnerability and its consequences for the patient and treatment regimen. Characteristics found in the plaque should be considered in the context of the risk factors, as these can influence the process of plaque formation. Individual risk factor management might be the holy grail in optimal reduction in risk for future cardiac events following coronary revascularization.
